# P-1595. Perceived Individual Risk of COVID-19 Mirrors Community Risk: Analysis of Testing Sites in West Virginia

**DOI:** 10.1093/ofid/ofaf695.1774

**Published:** 2026-01-11

**Authors:** Brad Price, Timothy Dotson, Gordon S Smith, Sherri Davis, Sally L Hodder

**Affiliations:** West Virginia University, Morgantown, West Virginia; West Virginia Clinical and Translational Science Institute, Morgantown, West Virginia; West Virginia University, Morgantown, West Virginia; West Virginia Clinical and Translational Science Institute (WVCTSI), Morgantown, West Virginia; West Virginia University School of Medicine, Morgantown, West Virginia

## Abstract

**Background:**

There are often large variations in individuals’ perceived vs. actual community risk of contracting infectious diseases. As part of a study to better understand attitudes and behaviors regarding testing and vaccination for COVID-19, we sought to examine factors related to differences between a person’s perceived risk vs. actual community risk and association of a positive SARS-CoV-2 test result.

**Table 1:**
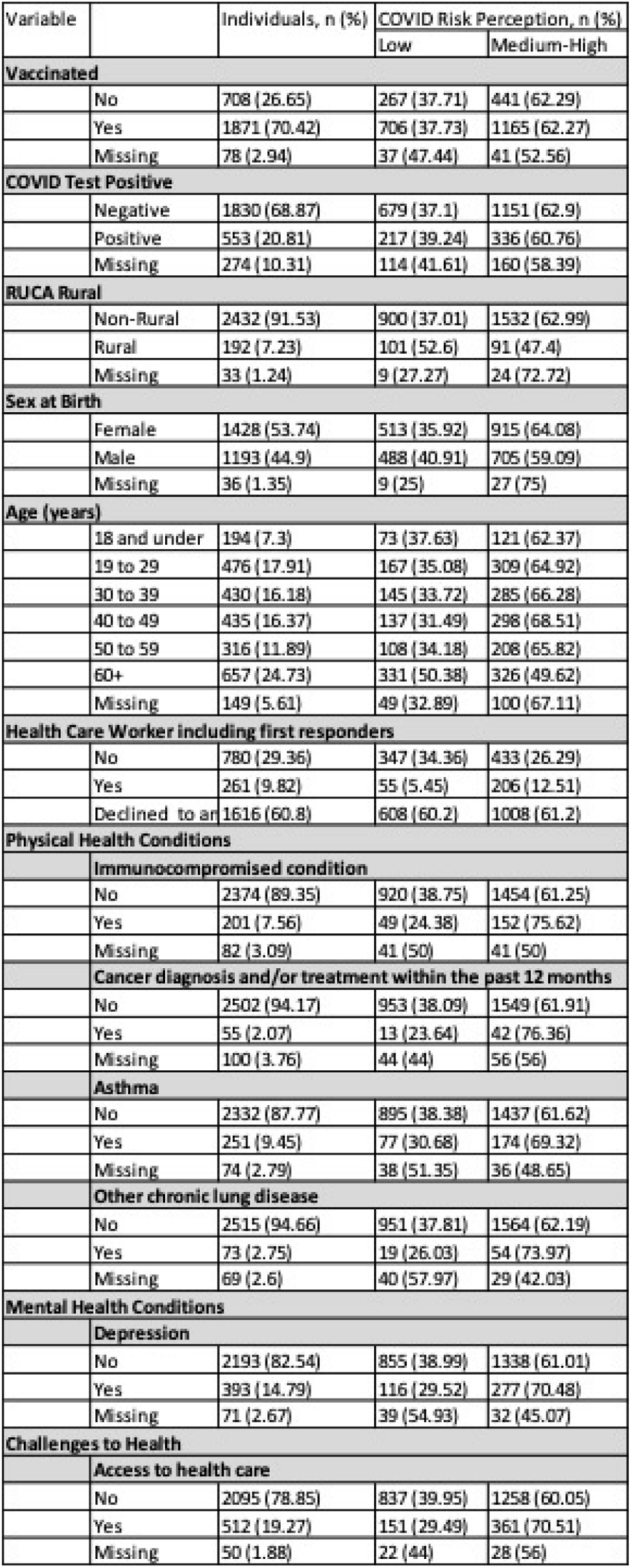
Demographics and Clinical Characteristics A breakdown of our study section by outcome response type arcross covariates.

**Table 2:**
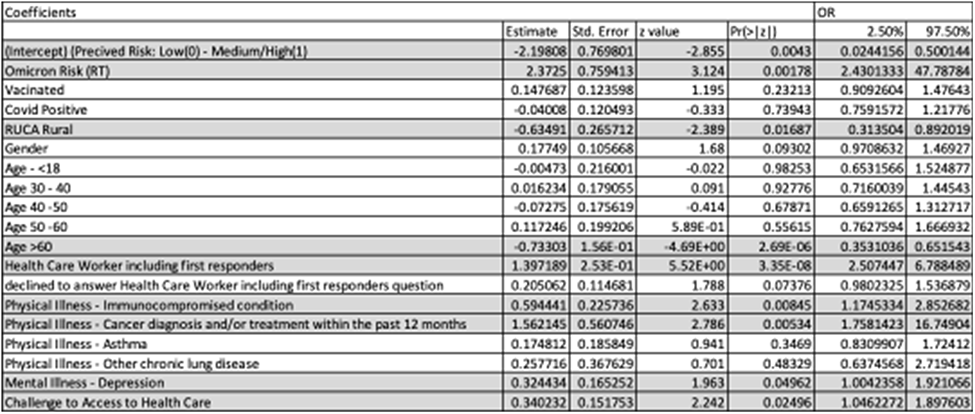
Modeling Results Across Groups

Results of our logistic regression model where perceived risk (Low=0, Med/High=1). Gray rows indicate statistically significant covariates at the p<0.05 level.

**Methods:**

As part of NIH’s RADx-UP program, surveys were conducted May 2022 to October 2023 among persons seeking COVID-19 testing. The question, *What do you think your personal level of risk is for getting sick from COVID-19,* assessed perceived COVID-19 risk. COVID test results were linked to individuals. Actual risk was measured by county level reproduction number, Rt. Logistic regression was used to examine differences between perceived and actual risk, accounting for other demographic factors, vaccination status, test result, and rurality.

**Results:**

Among 2657 respondents (surveys and tests), 90% conducted in 2022, 38.0% rated perceived risk as low and 62.0% as medium/high(Table 1).

Logistic regression comparing perceived and actual risk showed that overall, individuals’ perceived risk increases as their actual community (Rt) increases (p= 0.0043). Rural residents had significantly lower perceived risk than actual risk (p= 0.0169), as did persons 60+ years of age (p= < 0.0001). Those individuals estimating higher personal perceived risk were healthcare workers, immunocompromised persons, and those with a cancer diagnosis within the past 12 months. Table 2 provides full details of the analysis and adjusted odds ratios.

**Conclusion:**

When controlling for individual characteristics, individuals' perceived risk increases with community risk as measured by Rt. Rural residents underestimate their risk. Persons at high risk of COVID-19 and complications (e.g., immunocompromised) and healthcare workers are more aware of their risk for contracting COVID-19. These data are critically important for planning and resource allocation during future epidemics.

**Disclosures:**

Sally L. Hodder, M.D., Gilead Sciences: Advisor/Consultant

